# Effect of Chronic Ankle Sprain on Pain, Range of Motion, Proprioception, and Balance among Athletes

**DOI:** 10.3390/ijerph17155318

**Published:** 2020-07-23

**Authors:** Ahmad H. Alghadir, Zaheen A. Iqbal, Amir Iqbal, Hashim Ahmed, Swapnil U. Ramteke

**Affiliations:** 1 Rehabilitation Research Chair, College of Applied Medical Sciences, King Saud University, Riyadh 11433, Saudi Arabia; aha@ksu.edu.sa (A.H.A.); physioamir@gmail.com (A.I.); 2 Department of Rehabilitation Sciences, College of Applied Medical Sciences, Najran University, Najran 1988, Saudi Arabia; hahasan@nu.edu.sa; 3 Department of Musculoskeletal & Sports Physiotherapy, Sancheti Institute College of Physiotherapy, Shivaji Nagar, Pune 411005, Maharashtra, India; swapnil.com@gmail.com

**Keywords:** ankle joint, sprain, balance, Y-balance test, sport performance, musculoskeletal injury, rehabilitation, prevention and promotion of the health

## Abstract

*Background*: Ankle sprains are common among physically active individuals, especially among athletes. Majority of those who suffer ankle sprains have residual symptoms including pain, episodes of giving way, compromised proprioception and neuromuscular control, and re-injury leading to chronic ankle instability. The aim of this study was to see the effect of chronic ankle sprain on pain, range of motion, proprioception, and, static and dynamic balance among athletes. *Methods*: A total of 80 athletes, aged 18 to 25 years, involved in track-and-field sports were invited to participate in this study. They were divided in two groups. Athletes with history of grade 1 or 2 ankle sprain on either side requiring medical care who reported at least three episodes of ankle giving way in past 12 months were included in group A. An equal number of healthy athletes without any history of ankle sprain or injury in the lower limbs in the past one year matched by sex, age, height, weight, and limb dominance, were included in group B (control). *Outcome measures*: Participant’s pain, range of motion, proprioception and balance (static and dynamic) was measured using visual analog scale, half circle goniometer, degree of foot position sense, single leg stance time and Y-balance test respectively. *Results*: Although there were no differences in the active ankle joint range of motion (*p* > 0.05) in comparison to the control group, athletes with chronic ankle sprain reported mild pain and statistically significant (*p* < 0.05) deficits in foot proprioception, static and dynamic balance. *Conclusions*: Deficits in foot proprioception, static and dynamic balance even one year after the ankle sprain could be the reason for limitations in the dynamic defense system of the joint that predisposes to recurrent injury and instability. It is essential to understand the normal clinical course and risk factors for athletes who sustain sprain before devising a long term comprehensive rehabilitation program that focuses on mechanical and functional insufficiencies in order to improve their functional performance and prevent the risk of recurrent sprain.

## 1. Introduction

Sprains in the ankle joint are more common in physically active individuals, especially athletes where it accounts for at least 14% of all emergency hospital visits [1,2,3,4]. It has also been speculated that this figure could be higher as 50% of those who sustain an ankle injury do not report it or go to hospital for treatment [5,6]. In the acute phase, besides loss of range of motion (ROM) patients also have pain, swelling, muscle weakness, and deficit in postural control [2,7]. Research shows that at least 73% of individuals who suffer ankle sprain develop residual symptoms like pain, giving way, and compromised proprioception and neuromuscular control that increases the chances of re-injury and development of chronic ankle instability (CAI) [8,9]. History of multiple sprains and feeling unstable/giving way is referred to as CAI. Persistence of such instability over time increases the risk of articular damage and developing osteoarthritis [10]. The cost of treating and preventing such injuries is very high that has a major effect on the athlete’s training and return to play [11,12].

Investigators have reported that episode of previous injury, loss of ankle dorsiflexion, and balance deficit are strong predictor of recurrent ankle sprain in individuals with CAI [13,14,15,16]. Despite the associated costs, only few researchers have examined the effectiveness of preventive measures for ankle sprain [17,18]. Although static balance deficit has been widely reported in patients with CAI, fewer studies have studied dynamic balance deficit among them [19]. Similarly, fewer studies have examined the efficacy of proprioceptive training particularly in sportsmen [20]. Further investigations are required to establish the link between deficits in ROM, proprioception, and balance in chronic ankle sprain patients and development of CAI when they return to sports.

This study was conducted to see the effect of chronic ankle sprain on pain, ROM, proprioception, and, static and dynamic balance among athletes. We hypothesized that in comparison to their healthy counterparts, athletes develop CAI after return to sport due to deficits in ROM, proprioception, and balance.

## 2. Materials and Methods

### 2.1. Subjects

Convenience sample of 80 athletes involved in track-and-field sports, aged 18 to 25 years, were invited to participate in this study. Subjects were divided in two groups, A and B (control). Athletes with history of grade 1 or 2 ankle sprain on either side requiring medical care who reported at least three episodes of ankle giving way in past 12 months were included in group A. Talar tilts and anterior drawer’s tests were used to confirm involvement of anterior talofibular and Calcaneofibular ligaments respectively [21]. Athletes were excluded if there was any associated fracture or injury in the body, grade 3 sprain requiring surgical intervention, inadequate strength in lower limb or any vestibular or balance disorders. Equal number of healthy athletes matched for age, height, sex, weight, and limb dominance without any history of lower limb sprain in the past one year were included in group B.

Subjects preferred leg for kicking the ball was regarded as their dominant limb [22]. The limb that suffered ankle sprain was referred as affected limb. Limb length was measured as the distance from the most inferior aspect of anterior superior iliac spine to the most distal portion of lateral malleolus in supine position [23]. All subjects were allowed to continue their training and games during the study.

### 2.2. Outcome Measures

Participant’s pain, ROM, proprioception and balance (static and dynamic) were measured as described below. In group A, all measurements were done on affected limb that was matched for participants of group B.

- *Pain*: Pain was assessed using the Visual Analog Scale (VAS) [24]. Participants had to mark their pain on a 10 cm line with the two ends representing ‘no pain’ (0) and ‘worst possible pain’ (10). Distance (in cm) from the ‘0′ end was used a numerical index of the severity of the pain.
- *Active ROM*: Active dorsiflexion, plantarflexion, inversion and eversion ROM was measured using a standard hand-held half circle goniometer in supine lying position [25,26]. For measuring dorsiflexion and plantarflexion, proximal arm of the goniometer was placed parallel to lateral midline of the fibula, fulcrum was placed over the lateral aspect of the lateral malleolus, and distal arm was placed parallel to the lateral aspect of the fifth metatarsal. For inversion and eversion, proximal arm of the goniometer was placed parallel to the anterior midline of lower leg, fulcrum was placed over the lateral aspect of the 5th metatarsal heal, and distal arm was in line with the plantar aspect of the 1st to 5th metatarsal heads.
- *Proprioception*: Degree of foot position sense was used to record proprioception in the ankle [27,28,29]. Foot position sense was recorded by placing the ankle joint on non-affected side in some degrees of dorsiflexion or plantarflexion and asking athletes to match the position with the affected ankle with their eyes closed. Angle of difference in the position of ankles was noted in degrees.
- *Static balance*: Static balance was assessed using the single leg stance time while eyes open and closed [30]. It measures time (in sec) taken by an individual to maintain their base of support with minimal movement while standing on single leg.
- *Dynamic balance*: Y-balance test (YBT) was used to measure dynamic balance (Move2Perform, Evansville, IN, USA) [31,32,33,34,35]. It measures the reach distance in anterior, posteromedial, and posterolateral directions. Before the actual data collection participants were made to perform practice trials to accustom with the procedure. They were asked to stand barefooted on the affected leg and reach the indicator as far as they could by using the other leg and return to the starting position without losing balance. Reach distance was recorded to the nearest 0.5 cm. The trial was discarded if participants failed to return to the starting position without losing balance or they kicked the indicator. Three trials were recorded in each direction in the random order and the mean was used for data analysis. Reach distance was normalized by dividing by limb length and multiplying by 100 [36].

### 2.3. Statistical Analysis

Data was presented as mean and standard deviation (*SD*) and analyses were done using Graph-Pad Instat 3.0 (GraphPad Software, San Diego, CA, USA). Normality was analyzed using the Kolmogorov-Smirnov test. Independent *t*-tests were used to test the differences in age, height, and weight between the groups. An independent-samples t-test was used to compare the pain, active ROM, proprioception and static balance between the groups. Parametric analysis of variance using Bonferroni multiple comparisons test was conducted to test the difference in dynamic balance between the groups. Differences were considered significant when *p* values of less than 0.05.

### 2.4. Ethics Approval and Consent to Participate

All subjects were informed about the aims and procedures of the study and written informed consent was obtained. This study was approved by the Rehabilitation research review board for ethics according to the Declaration of Helsinki (approval number: RRC-2019-054).

## 3. Results

After considering the criteria for subject inclusion and exclusion, 30 athletes each were included in groups A and B. Demographic data are presented in Table 1. 

Mean differences in age, height and weight between the groups A and B were 0.7 years, 0.9 cm and 2.76 kg respectively. There were no significant differences between the two groups for age, height, weight and BMI (*p* > 0.05).

### 3.1. Comparison of Pain, Active Rom, and Proprioception between the Groups

Athletes of group A reported mild pain (mean VAS score 3.90 cm) at the time of assessment, while in group B there were no reports of pain. Mean differences in active dorsiflexion, plantarflexion, eversion, and inversion ROM between the groups A and B were −1.54, −1.84, −1.3, and −5.06 degrees respectively (Table 2). There were no significant differences between the groups for active dorsiflexion, plantarflexion, eversion, and inversion (*p* > 0.05). The mean difference in ankle dorsiflexion/plantarflexion position sense was significantly higher in group A (3.46 degrees) as compared to group B (1.25 degrees), *p* < 0.05.

### 3.2. Comparison of Static and Dynamic Balance between the Groups

As compared to group A (8.40 sec), single leg stance time values were significantly higher in group B (10.50 sec) during eyes open (*p* < 0.05). During eyes closed also it was significantly higher in group B (9.95 sec), as compared to group A (6.85 sec), *p* < 0.05. 

Mean difference in normalized reach distance (%) values between the groups A and B were −2.73, −9.81 and −5.72 in anterior, posteromedial and posterolateral directions respectively. As compared to group A, they were significantly higher in group B in all 3 directions (*p* < 0.05) (Table 3).

## 4. Discussion

This research was done to see the effect of chronic ankle sprain on pain, ROM, proprioception, and, static and dynamic balance among athletes. Results show that in comparison to their healthy counterparts, athletes with chronic ankle sprain reported mild pain and statistically significant deficits in foot proprioception, static and dynamic balance. However, there were no significant differences in the active ankle ROM between the groups. Besides statistically significant differences, it is important to analyze clinically significant differences as well.

After gradual improvement in the initial symptoms most of the patients who seek medical care do not go for complete rehabilitation program [37]. Restored physiological ROM one year after the injury despite residual joint dysfunction, as seen in our study, may be one of the reason for early return to sports without undergoing complete rehabilitation. This results in recurrent ankle sprains, prolonged disability, overuse injuries, and development of CAI and early osteoarthritis [38,39,40,41,42].

Two theories have been traditionally postulated for the cause of CAI: mechanical (MAI) and functional (FAI) ankle instability [42,43]. Individually, these two theories do not describe the full spectrum of CAI and form overlapping pathological contributions [42]. Interactions and relationships between MAI and FAI help to describe various aspects of CAI. Anatomical changes including pathologic laxity, impaired joint mechanics, synovial changes, and the development of degenerative joint disease, which may occur individually or in combination, as a result of initial injury leads to MAI. [44] These results in insufficiencies in the joint complex that further predispose instability. Injury to the ankle joint ligaments leads to adverse changes in the neuromuscular system that leads to FAI [45]. This further results in loss of dynamic support to the joint, impaired balance and proprioceptive deficits attributed to damaged articular mechanoreceptors in the ligaments and impaired neuromuscular control [46].

Studies have reported that MAI in the ankle is not restored up to at least 6–12 weeks after an ankle sprain and most of the patients continue to present mechanical laxity and complaints of instability, decreased function, pain, and/or swelling up to one year after injury [39,47,48,49,50,51,52]. This is more common among athletes as they tend return to sports without undergoing complete rehabilitation. Early and complete rehabilitation after sprain should be reinforced before return to the field as incomplete rehabilitation make the ankle more susceptible to re-injury [53]. The clinicians should convince patients with an ankle sprain to undergo complete rehabilitation despite improvement in the initial symptoms. In order to reduce the incidence of recurrent sprains, rehabilitation program should address components of both mechanical and functional instability. Comprehensive rehabilitation programs including proprioceptive, neuromuscular control, and balance training have been shown to significantly reduce the risk of recurrent ankle sprains [54,55].

Results of this study shows deficits in both static and dynamic balance and other parameters one year after the ankle injury causing sprain. This shows that although symptoms of acute phase have already to resolved, deficits occur due to establishment of various long-term factors [56,57]. In comparison to static balance, ability to maintain dynamic balance is a better indication of functional ability especially in physically active people like sportsmen [58,59]. Fewer studies have studied dynamic balance in patients with ankle sprain among them, [19] and this is one of the few studies that have used YBT to assess dynamic balance among athletes with chronic ankle sprain. Chronic sprain has been shown to have central effects on the sensorimotor system that leads to development of balance and posture control deficits [60,61,62]. In addition to structure of the ligaments, various mechanoreceptors in the joint are also damaged following sprain, [63] that provide the sense of joint position and movement, further providing feedback about joint pressure and tension [45]. This information is further processed with the vestibular and visual systems through afferent nerve fibers for posture control and coordination [64]. When this information is altered after injury it leads to development of joint instability and functional impairments, increasing the chances of recurrent sprain [65,66].

The results of this study also show deficits in ankle proprioception in patients with chronic ankle sprain. This is supported by previous studies that compared the joint position sense error as active replication of joint angles or measures of kinesthesia in patients with ankle sprain showed significantly greater error as compared to uninjured side or healthy controls [67,68,69,70,71]. Impairments in postural control are due to a combination of deficits in neuromuscular control and proprioception caused by the joint injury that further limits the dynamic defense system of the joint and predisposes recurrent injury and instability [60,72,73,74,75]. Joint position sense, as considered in this study, is the ability to recognize the joint location that influences joint stability and body alignment. It is suggested that increased joint stability following proprioceptive exercises may also affect dynamic balance [76,77]. Although the effect of proprioceptive exercises on ankle joint has been subject of many studies, further research is required to study the effect of more common treatment strategies in order to improve the clinical outcomes among athletes following the ligament injury at the ankle.

As the pain and swelling decrease after initial intervention, athlete should begin a progressive therapeutic exercise program focusing on improving overall lower extremity function [78,79]. Functional outcome measures that integrate strength, ROM, proprioception, and neuromuscular control, should be included in assessment following an ankle sprain [21,80]. Although it has been reported that a diverse and low-frequency exercise program can improve ankle instability [81], efficacy of a balance training program as a primary intervention for the prevention of ankle sprains among athletes has not been reported. Rehabilitation programs should also focus on strength and balance training of the uninvolved limbs and joints proximal to the ankle joint [57,82,83]. Balance exercises referred in most of the previous studies are very general and may not target the deficits athlete experience. It is essential to understand the risk factors and normal clinical course for athletes who sustain sprain before devising complete rehabilitation program [21], and further research is needed to improve the clinical outcomes of athletes who suffer ankle instability. Based on these findings a long term comprehensive rehabilitation program including neuromuscular control, strength, proprioceptive, and balance training should be devised that challenges the musculoskeletal, visual, somatosensory, and vestibular systems of the body that influences an athlete’s postural stability and control, and addresses arthrokinematic changes, pathologic laxity, and other mechanical insufficiencies in order to improve their functional performance and prevent the risk of recurrent sprain [3,53,54].

## 5. Limitations

This study didn’t comply with the International Ankle Consortium position statement [84]. We propose similar prospective study with a larger sample and considering broader characteristics including history of initial injury, treatment obtained, and the ratings of patient disability and function in the inclusion criteria to determine the efficacy of sports-specific training over balance training in decreasing the risk of re-injury at the ankle joint. Regarding static and dynamic balance tests, the support of the left and right lower limbs should also be matched by muscular strength and one-sided jump, etc.

## 6. Conclusions

Results of this study show that although there were no differences in the active ankle joint ROM, athletes with chronic ankle sprain reported significant deficits in foot proprioception, static and dynamic balance. This could be the reason for limitation in the dynamic defense system of the joint that predisposes recurrent injury and instability. It is essential to understand the risk factors and normal clinical course for athletes who suffer sprain before devising a long term comprehensive rehabilitation program that focuses on their mechanical and functional insufficiencies.

## Figures and Tables

**Table 1:** Demographic data of the athletes presented as Mean (SD).

Variables	Group A	Group B	*p*
Number	30	30	n/a
**Gender**			
Female	15	15	n/a
Male	15	15	n/a
**Ankle sprain**			
Grade I	17	n/a	n/a
Grade II	13	n/a	n/a
Age-years	21.40 (2.16)	22.10 (4.15)	0.062
Height-cm	169.70 (3.56)	170.60 (4.39)	0.059
Weight-kg	69.34 (12.68)	72.10 (13.24)	0.060
BMI-kg/m^2^	24.07 (5.54)	24.77 (6.93)	0.066
**Dominance**			
Right	28	28	n/a
Left	2	2	n/a
**Affected limb**			
Right	26	0	n/a
Left	4	0	n/a

n/a: not applicable ns: not significant.

**Table 2:** Comparison of pain, active range of motion (AROM), and proprioception between the groups: Mean (SD).

Variables	Group A	Group B	*p*
**Pain**			
Visual Analog Scale-cm	3.90 (0.76)	00 (00)	0.000 *
**AROM**-degrees			
Plantarflexion	40.61 (4.91)	42.15 (8.83)	0.058
Dorsiflexion	10.56 (3.67)	12.40 (6.80)	0.063
Inversion	30.92 (3.70)	32.22 (15.49)	0.055
Eversion	17.61 (1.35)	22.67 (5.69)	0.059
**Proprioception**			
Degree of foot position sense-degrees	3.46 (1.18)	1.25 (1.05)	0.010 *

ns: not significant; * Significant.

**Table 3:** Comparison of static and dynamic balance between the groups: Mean (SD).

Variables	Group A	Group B	*p*
**Static balance**: Single leg stance time-sec			
Eyes open	8.40 (1.24)	10.50 (0.95)	0.01 *
Eyes closed	6.85 (1.12)	9.95 (1.00)	0.02 *
**Dynamic balance**: Y balance test-percentage			
Anterior	73.63 (3.08)	76.36 (5.25)	0.04 *
Posteromedial	86.61 (3.98)	96.42 (9.51)	0.001 *
Posterolateral	82.78 (4.10)	88.5 (11.67)	0.01 *

* Significant.

## List of Abbreviations

ROM	Range of motion
CAI	Chronic ankle instability
VAS	Visual analog scale
YBT	Y-balance test
SD	Standard deviation
MAI	Mechanical ankle instability
FAI	Functional ankle instability
